# Type 2 diabetes mellitus in children: are we ‘treating’ them right?

**DOI:** 10.1186/1687-9856-2015-S1-P21

**Published:** 2015-04-28

**Authors:** Anuar Azriyanti, Zarina Yaakop, Jalaludin Mohammad Yazid, Harun Fatimah

**Affiliations:** 1 Department of Paediatrics, Faculty of Medicine, University Malaya, Kuala Lumpur, Malaysia

## Background

Incidence of Type 2 diabetes mellitus (T2DM) in children is rising alongside childhood obesity. In children, there is limited therapeutic options and tight blood glucose (BG) control may be challenging.

## Aims

To determine metabolic control and complications in children with T2DM seen in University Malaya Medical Centre (UMMC).

## Methods

Data on children with T2DM referred to and managed in UMMC from 2000 until 2013 were collected. Their body mass index (BMI and blood tests (HbA1c and Lipids) were compared at presentation to their latest clinic appointment. Treatment modalities and duration of follow up were documented. T2DM was dignosed if they had hyperglycaemia (Random BG > 11.8mmol/L or fasting BG > 7mmol/L or 2hpp OGTT >11.1mmol/L with low C-Peptide). Hypertension (HPT) if BP> 90th centile for age,sex and height. Dyslipidemia is considered if either triglycerides> 1.7mmol/L, cholesterol >5.2mmol/L, HDL< 1.03mmol/L or LDL> 2.50mmol/L. Non-alcoholic fatty liver disease (NAFLD) was confirmed with ultrasound, diabetic nephropathy (DN) if urine microalbumin>3.5 in boys , >4.5 in girls and diabetes retinopathy(DR) if reported by opthalmologist.

## Results

A total of 49 children with T2DM were seen, but only 37 had available data for analysis. Their age ranged from 7-17 years old at initial presentation. Forty nine percent (n=24) were boys. The mean duration for follow up was 3.6 years(0.2-10years).

**Table 1:** 

	At presentation	At last visit
Mean Age:	11.79 years old (7-17)	15.3 years old (8.9-22)

Mean Weight:	61kg	70.4kg

Mean BMI (SDS):	28kgm^2^ (+2.62)	28.5kgm^2^ (+1.91)

Mean WC:	97.0cm	94.3 cm

Mean HbA1c:	11.2% (7.4-16%)	9.7% (5.4-14.4%)

Metformin:	56% (21/37)	92% (34/37)

Insulin:	32%(12/37) ** mainly due to ketosis at diagnosis	32% (12/37) ** mainly due to poor BG control

Dsylipidemia	92% (22/24 screened)	87% (21/24 screened)

Fatty Liver:	Not screened at diagnosis	80% (8/10 screened)

Nephropathy:	Not screened at diagnosis	24% (9/37)

Hypertension	24% (9/37)	8% (3/37)

Retinopathy:	Not screened at diagnosis	1 had early changes of diabetes retinopathy

## Conclusions

This study revealed children with Type2DM had poor metabolic control with mean HBAc of 9.7% and early complications were already seen after 3.6 years of follow up.

